# Editorial: Crop domestication and selection: an evolutionary view affecting the development of agronomic traits

**DOI:** 10.3389/fpls.2025.1622204

**Published:** 2025-06-17

**Authors:** Bliss M. Beernink, Hong An, Ting Xiang Neik, Li Lei

**Affiliations:** ^1^ Lawrence Berkeley National Laboratory, U.S. Department of Energy Joint Genome Institute, Berkeley, CA, United States; ^2^ Bioinformatics and Analytics Core, University of Missouri, Columbia, MO, United States; ^3^ Department of Biological Sciences, Faculty of Science, National University of Singapore, Singapore, Singapore

**Keywords:** crop, domestication, selection, development, evolution, agronomic trait

Crop domestication – the evolution of wild plants for human use – is central to agriculture. Through artificial selection, humans have shaped traits like larger seeds and tastier fruits, a process known as the “domestication syndrome” (Alam and Purugganan, 2024). Modern crops reflect complex interactions with their wild progenitors (Palombo et al.). Studying domestication from an evolutionary perspective enhances our understanding of plant biology and informs crop improvement. This *Frontiers* Research Topic explores how crop domestication and selection have shaped key agronomic traits, and how this knowledge can guide future agricultural innovation, uniting studies from genomic origins to specific domestication traits to show where evolution and human innovation intersect.

## Evolutionary histories and genetic diversity of crops

A recurrent theme in this *Frontiers* Research Topic is the value of wild relatives and ancestral lineages in understanding crop domestication. Palombo et al. examined the locoto chile (*Capsicum pubescens)*, an Andean pepper with no known wild populations. Using genome-wide markers, the authors reconstructed *C. pubescens’* lineage, finding it closely related to two wild species (*C. cardenasii* and *C. eximium*). Their results suggest that the gene flow from wild relatives has significantly influenced the locoto pepper’s genetic diversity, highlighting domestication as a complicated, non-linear process shaped by ongoing exchange between wild and cultivated populations, introducing useful variation into the crop gene pool.

Xie et al. review wo under-domesticated “wild rice” species: *Zizania latifolia* (East Asian, used as a vegetable) and *Zizania palustris* (North American, used as a grain). Though long utilized as food, both have only recently seen systematic breeding. Genomic and omic tools, have elucidated these species’ origins and key agronomic traits such as yield, stress tolerance, and nutrient use. *Z. latifolia*, a perennial, offers sustainability advantages over annual *Z. palustris*. The review synthesizes decades of research on the edible history, economic value, and breeding advances in *Zizania*, noting how genetic insights are now accelerating the domestication of these crops into more productive and resilient varieties.

Wei et al. analyzed the genomic scale of selection in polyploid crops, specifically by examining the genomes of three tetraploid *Brassica* species – *B. napus* (rapeseed), *B. juncea* (mustard), and *B. carinata* (Ethiopian mustard) – to explore how selective pressures have shaped their evolution. Formed through hybridization of diploid ancestors these species show clear genomic signatures of selection, with 9,701 genes identified as being under positive selection. These genes were often shorter, tended to be single-copy or tandem duplicates, and enriched in key metabolic pathways like lipid biosynthesis in oil-rich *B. carinata.* This genomic analysis elegantly demonstrates how human selection leaves molecular signatures.

## Developmental traits shaped by domestication

Suganami et al. examine awnlessness in Japanese rice, revealing it as a modern breeding goal rather than an ancient domestication trait. Awned varieties were common until about a century ago, when awnless types were favored for easier harvest. Despite independent breeding efforts, all awnless lines share a mutation in the *OsEPFL1* gene, showing how a single genetic change was repeatedly selected. This study redefines awnlessness as a modern crop improvement, and underscores how quickly human selection can reshape plant development in response to agricultural needs.

In fruit tree crops, Wang et al. investigated controlling preharvest fruit drop in litchi (*Litchi chinensis*), a crucial yield related agronomic trait. Comparing a drop-prone cultivar (“Nuomici”) with a drop-resistant one (“Huaizhi”) authors found an early activation of cell-wall-degrading enzymes weakened fruit attachment in the drop-prone cultivar. The authors proposed a signaling pathway model linking hormone changes to cell separation processes resulting in early fruit drop​. Like the rice awn study, this work demonstrates how domestication and selective breeding have fine-tuned plant development to better meet agricultural needs by suppressing or retaining certain organs.

Jareczek et al. reviews how domestication has transformed cotton fiber from the wild cotton’s short fibers to domesticated cotton’s long, spinnable fibers that have been vastly extended and thickened through human selection and has revolutionized textiles. This review describes the integration of developmental and evolutionary perspectives on the domestication of cotton and highlights how selection reshapes cellular development. It also points to future possibilities: understanding the genes and regulatory networks behind fiber growth may enable further improvements in fiber crops or even inspire the bioengineering of novel plant fibers.

## Emerging crops and future perspectives

As we deepen our understanding of domestication, we can use that knowledge to continue improving underutilized crops and tackle modern agricultural challenges. Hagelthorn and Fletcher explore pennycress (*Thlaspi arvense*), a weedy mustard being domesticated as a winter oilseed cover crop. Focusing on the *CLAVATA3/ESR-related* (*CLE*) gene family, which regulates plant growth and organ size. They identified 27 genes with seed specific expression patterns, and found that modifying *CLE* peptides could promote domestication traitslike compact growth or altered branching. This research provides a genomic toolkit for pennycress improvement, showcasing how targeted gene modification can accelerate domestication.

In an era of climate change and resource constraints, one critical trait is nutrient use efficiency. By mapping a 605-gene nitrogen utilization network, Li et al. tackled this issue in *Brassica napus* (canola). The authors identified nine root-expressed genes activated under nitrogen deficiency, making them prime targets for improving nitrogen stress tolerance​. Cross-species comparisons revealed that the nitrogen-utilization network is ancient and conserved, indicating that optimization of existing networks could enhance nitrogen efficiency. This knowledge offers a roadmap for breeding canola varieties that that maintain yield with less fertilizer, aligning modern goals with the ongoing domestication.

In conclusion, the articles in this Research Topic collectively underscore that crop domestication is an ongoing and dynamic process shaped by both evolution and human innovation. From chili peppers in the Andes to wild rice in far-flung wetlands, each study illuminates how crops have been tailored to human needs – and how, in turn, they reveal fundamental principles of plant development, genetics, and evolution. Viewing crop traits through an evolutionary lens not only explains the past but also guides the future, offering tools to improve existing crops and domesticate new ones. As agriculture faces growing challenges, these insights into crop domestication and selection are and will be essential for building more resilient and sustainable food systems.
